# Impact of immune function and white matter integrity on cognitive function in patients with bipolar disorder

**DOI:** 10.3389/fimmu.2026.1829931

**Published:** 2026-07-31

**Authors:** Zhipeng Wang, Tingting Xie, Yumei Wang, Xihua Jia, Xiaobo Wang

**Affiliations:** 1 Department of Ultrasound, the Third Medical Center of Chinese PLA General Hospital, Beijing, China; 2 Institute of Brain Science and Brain-Inspired Research, Shandong First Medical University &Shandong Academy of Medical Sciences, Jinan, Shandong, China; 3 Departmentof Psychology, Shandong Provincial Hospital Affiliated to Shandong First Medical University, Jinan, Shandong, China; 4 Department of Oncology, The First Central Hospital of Baoding, Baoding, Hebei, China; 5 Department of Clinical Psychology, The First Central Hospital of Baoding, Baoding, Hebei, China

**Keywords:** bipolar disorder, cognitive function, inflammatory factors, lymphocyte subpopulations, white matter structure

## Abstract

**Introduction:**

Cognitive impairment significantly impacts social function in Bipolar disorder (BD). This study explores the link between immune markers, white matter microstructure, and cognitive function in depressed BD patients enhance early diagnosis and treatment.

**Methods:**

The study included 52 depressed BD patients and 40 healthy controls(HC), collecting data on inflammation, lymphocyte subpopulations, MRI scans, and cognitive tests, using Spearman correlation and machine learning for comparison.

**Results:**

This study found that the CD4+ T cell count (*P* = 0.002) and the percentage of regulatory T cells (*P* = 0.003) in depressed BD patients were significantly higher than those in the HC. Additionally, the expression levels of IL-6, IL-8, and TNF-α in the BD group were significantly higher than those in the HC (*P* values of 0.020, 0.012, and 0.029). Analysis of the fractional anisotropy (FA) and mean diffusivity (MD) of white matter revealed that depressed BD patients had significantly lower FA values in multiple brain regions compared to HC, including the right posterior limb of the internal capsule (*P* = 0.009), right medial thalamic tract (*P* = 0.017), right posterior thalamic radiation (*P* = 0.046), right sagittal stratum (*P* = 0.041), and left sagittal stratum (*P* = 0.038). Additionally, cognitive function test results indicated that the Go/No-Go accuracy of depressed BD patients was significantly lower than that of HC (*P* = 0.027); the ODD reaction time of depressed BD patients was significantly longer than that of HC (*P* = 0.04). Correlation analysis showed that IL-6 was weakly correlated with the Go/No-Go accuracy difference (r=-0.044, P = 0.027). the MD values of the left superior corona radiata and left superior frontal occipital tract were negatively correlated with the difference in Go/No-Go accuracy (r=-0.220 and r=-0.517). Results from the support vector machine (SVM) model indicated that the Stroop task (β=1.155 ± 0.056), the inflammatory factors IL-6 (β=1.073 ± 0.069) and TNF-α (β=1.071 ± 0.062) demonstrated the highest discriminative power in distinguishing BD depression.

**Conclusion:**

This study suggests that neuroinflammation and white matter structure play pivotal roles in the pathogenesis of BD with a depressive episode. These findings advance the understanding of the immunological and neuroimaging underlying mechanisms in BD with a depressive episode and highlight potential drivers of cognitive impairment.

## 1 Introduction

BD is a recurrent illness that typically manifests in late adolescence or early adulthood, with over 75% of patients showing clinical features of the disorder before the age of 25 (1, 2). The recurrence rate is as high as 90% (3). The chronic course of BD is one of the main causes of disability among young people and the working-age population (4), profoundly impacting the daily lives of patients and imposing a significant economic burden on families and society (5).

Cognitive impairment is one of the core features of BD, which impairs patients’ ability to maintain optimal social and occupational functioning, compromising the quality of life, and persisting throughout all stages of the illness, including euthymic states (6–8). Research indicates that cognitive deficits in depressed BD patients may be secondary to immune dysfunction. Studies using animal models demonstrate that the immune system plays a pivotal role in maintaining the long-term integrity of cognitive function (9). It has been reported that inflammatory cytokine levels increase during periods of depression, mania, and mood stabilization (10), and this increase alters monoamine levels, thereby affecting cognitive and emotional functioning (11). Inflammatory immune responses are considered a major factor contributing to alterations in brain microstructure and impaired neural plasticity (12), which collectively underlie cognitive impairment (13).

Neuroimaging techniques are fundamental in elucidating neurocognitive functions, particularly through the use of diffusion tensor imaging (DTI) to explore the relationship between cognitive functions and white matter connectivity. This technique has garnered increasing attention as it assesses white matter microstructure by quantifying the magnitude and directionality of water molecule diffusion (14). DTI models tensors to define and quantify the diffusive motion of water molecules within axonal fibers. The most commonly used diffusion metrics are FA, which describes the degree of anisotropy in brain tissue and reflects the extent of diffusion along a single direction of the axon (15), and MD, which describes the overall diffusion of water molecules and reflects changes in the extracellular space and axonal loss (16). These indicators serve as indicators of the integrity and microstructural changes of cerebral white matter (WM). DTI studies have demonstrated that patients with BD have significantly lower FA values in various regions of the corpus callosum (CC) compared to HCs, particularly within the body and posterior regions of the corpus callosum (17). Cognitive impairments in BD are closely related to white matter deficits, particularly deficits in sustained attention, which is directly linked to the integrity of the white matter in the corpus callosum (18). These studies suggest that white matter microstructural alterations in BD may serve as biological markers for cognitive dysfunction.

Existing studies have shown that neuroinflammation affects the microstructure of WM (19), yet its underlying mechanisms remain unclear. A study using the Tract-Based Spatial Statistics (TBSS) method, including 31 patients diagnosed with bipolar disorder (BD) and depression, found that inflammatory cytokines were associated with higher MD and reduced connectivity, suggesting that the activated inflammatory response system may promote the pathophysiology of BD by obstructing the structural connectivity of key cortical-limbic networks (20). Studies indicate that elevated inflammatory markers are associated with changes in white matter microstructure measured by DTI (21).Moreover, specific inflammatory markers such as C-reactive protein (CRP) and tumor necrosis factor-alpha (TNF-α) are implicated in cognitive dysfunction in BD (22).Therefore, exploring the relationships among white matter, inflammatory markers, and cognitive function in BD has significant clinical and theoretical implications.

This study aims to conduct a multidimensional analysis of white matter microstructure and immune characteristics in patients with depressed BD by combining DTI and immunohistochemistry methods. This study investigates on exploring the relationships between peripheral blood inflammatory factors, including C-reactive protein (CRP) and lymphocyte subsets, and cognitive function in BD patients. By comparing DTI indicators (FA, MD) between depressed BD patientspatients and HC, this study aims to reveal potential white matter damage in the brains of BD patients and its association with cognitive function. This study employs a multi-omics approach to elucidate the pathological mechanisms of BD, focusing on the relationships among immune-inflammatory responses, white matter structure, and cognitive function. This study is expected to provide new biomarkers and therapeutic targets for clinical practice, thereby laying a pathophysiological foundation for improving patient prognosis and quality of life.

## 2 Materials and methods

### 2.1 Participant profiles

This study was conducted at the Clinical Psychology Department of Baoding First Central Hospital, in Hebei Province, China.The study included 52 depressed BD patients —and 40 HC matched for age, all recruited from our hospital between April and December 2024. Enrolled patients will undergo magnetic resonance imaging (MRI) examinations, which were performed following strict protocols to ensure equipment functionality and procedural adherence. Peripheral blood samples were collected using sterile techniques to ensure quality and safety. Cognitive function assessments were conducted using standardized instruments to ensure evaluation accuracy and reliability.

The inclusion criteria were as follows: 1) aged between 15 and 25 years, 2) diagnosed with BD according to the 10th Revision of the International Statistical Classification of Diseases and Related Health Problems (ICD-10) (23), 3) no prior receipt of any form of treatment, including medication and psychotherapy, for at least two weeks before study initiation. The exclusion criteria were as follows: 1) individuals with other mental disorders, such as depression or schizophrenia, 2) individuals with conditions that may affect inflammatory markers, including acute inflammatory illnesses and metabolic syndromes like autoimmune diseases, infections, blood disorders, diabetes, and heart failure, 3) those with a current or history of substance abuse or dependence within the past six months. Healthy control subjects were required to meet the following criteria: 1) aged between 15 and 25 years, 2) confirmation via Structured Clinical Interview (SCID) of the absence of mental disorders, 3) free from conditions that could affect inflammatory markers, including acute inflammatory illnesses and metabolic syndromes such as autoimmune diseases, infections, blood disorders, diabetes, and heart failure.

This study received approval from the Institutional Review Board of the hospital (Approval No. 2023083) prior to data collection and was performed in accordance with the Declaration of Helsinki and its subsequent revisions. Written informed consent was obtained from all participants, ensuring their understanding of the study’s purpose and procedures. Furthermore, all participants were informed that they were free to withdraw from the study at any time without providing a reason. All data collected were handled with strict confidentiality and anonymity to protect the participants’ privacy.

### 2.2 Blood sample collection

Two 5-mL blood samples were collected from the peripheral veins of HCs and patients with BD into EDTA anticoagulant tubes. The blood samples were immediately centrifuged at 3000 g for 15 minutes. Following centrifugation, the upper plasma layer was carefully transferred to pre-labeled cryovials without disturbing the cellular pellet. The plasma samples were stored at -80 °C until all sample collection was completed. Subsequently, plasma levels of inflammatory factors, including IL-6, IL-8, CXCL2, CCL2, TNF-α, G-CSF, and CRP, were quantified using enzyme-linked immunosorbent assays (ELISA). The second blood sample was used for lymphocyte subset analysis.

#### 2.3.1 ELISA experiment

Peripheral venous blood samples collected from all participants were analyzed using enzyme-linked immunosorbent assay (ELISA) to detect IL-6, IL-8, CXCL2, MCP-1, TNF-α, G-CSF, and CRP, and the differential expression between depressed BD patients and HC was compared.The assays utilized ELISA kits supplied by provided by Hubei Youpin Biotechnology Co., Ltd, following the procedures outlined by the manufacturer. All kit performance: the linear regression of the standard correlates with the expected concentration correlation coefficient R, which is greater than or equal to 0.9900. The sensitivity of this assay is 1.0 pg/ml.

#### 2.3.2 Flow cytometry analysis

Before use, the flow cytometer should undergo calibration and confirmation of normal working status. After anticoagulation treatment, the blood sample is diluted with PBS to 1:10–1:20.Follow the manufacturer’s instructions and add fluorescently labeled antibodies (CD3-APC-H7 [Catalog Number: 663490], CD4-PE-Cy7 [Catalog Number: 663493], CD8-APC-Cy7 [Catalog Number: 565309], CD19-APC [Catalog Number: 340721], CD16+CD56-PE [Catalog Number: 340042], and CD127-PE [Catalog Number: 560822]) at the recommended concentrations and incubated at 4 °C for 30 minutes, all of which are from Becton, Dickinson and Company. Following two washes with PBS, the cells are resuspended in PBS. Data are collected using the calibrated flow cytometer configured with the appropriate lasers and fluorescence channels. Record the counts of CD3+ T, CD4+ T, CD8+ T, CD19+ B, CD56+NK cells and the percentage of Treg cells among CD4+ T cells., and flow cytometry software is used to generate cell population distribution plots and perform statistical analysis.

#### 2.3.3 Cognitive function assessment

This study employed various assessment instruments to evaluate the cognitive functions of the subjects. Executive functions encompass a range of higher cognitive processes that guide, control, and manage cognitive, emotional, and behavioral responses. Executive functions are widely recognized as comprising the following three core areas:

1. Working memory: The spatial location memory task is a cognitive paradigm that assesses spatial working memory ability, aiming to test participants’ accuracy and persistence in remembering a series of randomly presented stimulus locations (24). N-back task assesses working memory capacity, Measure working memory performance through reaction time and accuracy to assess an individual’s judgment ability in working memory tasks (25).
2. Inhibitory control: The Stroop task is used to assess inhibitory control ability by quantifying the strength of inhibitory control through the difference in reaction time and accuracy between congruent and incongruent conditions of color words (26). Go/No-Go task:During the testing process, it is required to respond to certain stimuli while not responding to others, in order to quantify an individual’s control and decision-making ability in response inhibition tasks (27). Stop-Signal task:Assessing an individual’s ability to stop after initiating a behavior (28).
3. Cognitive flexibility: The More-odd shifting task measures cognitive flexibility by comparing the difference in reaction time and accuracy between shifting and non-shifting conditions (29).

The experimental protocol is as follows:

Participants were tested in a quiet environment to minimize external distractions and facilitate optimal concentration during the test. Administer the test tasks sequentially in accordance with the standard operating procedures of the selected assessment tool, documenting each participant’s reaction time and accuracy. Subsequently, analyze the collected data statistically using appropriate methods to evaluate executive function performance and compare results between the experimental and control groups.

#### 2.3.4 MRI data acquisition

1. Conduct a detailed health assessment of the subjects before scanning to ensure there are no metal implants or other MRI contraindications.
2. Place the subjects on the scanning bed of the 3T Siemens Skyra scanner, ensuring their heads are fixed to reduce motion artifacts.
3. Adjust the head fixation device according to the size of the subjects’ heads to ensure comfort and stability.
4. Set the scanning parameters. This study uses a three-dimensional magnetization prepared rapid acquisition gradient echo (3D MPRAGE) sequence to acquire T1-weighted structural images. A planar echo (EPI) diffusion-weighted sequence (ep2d_diff) combined with a bipolar diffusion scheme is used. The scanning parameters are as follows: 2D planar echo diffusion tensor imaging (EPI-DTI), TE = 92 ms, TR = 4300 ms, flip angle of 90 degrees, reference resolution and acquisition matrix both 128×128, slice thickness of 4 mm, inter-slice spacing of 5.2 mm, and phase encoding direction as column (COL). Generalized autocalibrating partially parallel acquisitions (GRAPPA) are used for in-plane parallel acceleration with an acceleration factor of 2. The diffusion sensitivity parameters include two b-values: b=0 s/mm² and b=1000 s/mm², with a total of 61 diffusion images collected (one direction for b=0 and sixty directions for b=1000).
5. Start the scanning procedure and monitor the scanning process to ensure no abnormalities occur while collecting imaging data.

#### 2.3.5 Image processing

This study used FMRIB Software Library (FSL) and MRtrix3 software to process all diffusion-weighted imaging(DWI) data, with the following specific workflow: First, the original digital Imaging and Communications in Medicine (DICOM) data was converted to Neuroimaging Informatics Technology Initiative (NIfTI) format using the dcm2niix tool, followed by a series of preprocessing steps: (1) Noise removal was performed using the dwidenoise module of MRtrix3, and then Gibbs ringing artifacts were corrected using the mrdegibbs module. (2) The eddy module of FSL was used for distortion correction due to eddy currents and head motion correction to reduce artifacts caused by motion and eddy effects. (3) Bias field correction was performed using FMRIB’s Automated Segmentation Tool (FAST) module to eliminate signal intensity inhomogeneity. After preprocessing, the DTIFIT tool of FSL was used to fit the diffusion tensor imaging model DTI, generating quantitative parameter images of FA, MD to standardize individual white matter microstructural characteristics.

To conduct standardized component analysis, the FA images were first linearly registered to T1-weighted structural images, and then the T1 images were registered to Montreal Neurological Institute (MNI) standard space using FMRIB’s Nonlinear Image Registration Tool (FNIRT), obtaining the transformation matrix from MNI space to individual FA space. This matrix was then used to transform the JHU white matter fiber bundle atlas to each subject’s FA space. Based on the transformed atlas, the average values of FA, MD, and S0 (non-diffusion weighted signal) were extracted from 50 regions of interest (ROI) and imported into SPSS for subsequent inter-group comparative analysis.

### 2.4 Machine learning and statistical analysis

#### 2.4.1 Machine learning

After statistical screening, a total of 19 feature variables were included for subsequent machine learning analysis, comprising 4 executive function indicators, 9 FA value indicators, 3 immune cells and 3 inflammatory factors. In the machine learning modeling process, we used Support Vector Machine (SVM) for classification, selecting a linear kernel function. To ensure data quality, we performed z-score standardization on all features and converted the class labels into factor variables. Considering the limitation of sample size, we employed the Leave-One-Out Cross-Validation (LOOCV) strategy for model validation, dividing the total sample into 92 folds, using 91 samples for training each time, with the remaining 1 sample for testing, and recording the predicted probabilities and true labels for each fold. To evaluate the classification performance of the model, the following metrics were calculated: Accuracy, Sensitivity, Specificity, Receiver Operating Characteristic (ROC) curve, and Area Under the Curve (AUC). All statistical analyses were completed using R software (version 4.1.0).

#### 2.4.2 Statistical analysis

Data processing and statistical analysis were performed using SPSS 27.0 statistical software. The Kolmogorov-Smirnov method was used for normal distribution testing. For normally distributed continuous data, the results are expressed as mean ± standard deviation (SD). Independent samples t-test was used for comparisons between two groups. If the data did not conform to a normal distribution, the median (interquartile range) was used for statistical description, and the Mann-Whitney U test was used for intergroup comparisons. Categorical data were expressed as frequency and percentage (%), with χ2 test used for within-group comparisons, and Spearman correlation analysis was employed. Correlation plotting analysis was conducted using GraphPad Prism. A two-tailed test was used, with *P* < 0.05 considered statistically significant.

## 3 Result

### 3.1 Characteristics of depressed BD patients and HC

This study recruited 92 participants, comprising 52 depressed BD patients and 40 HCs. **Table 1** presents demographic and clinical characteristics, including age, sex, Body Mass Index (BMI), education, and smoking status, for both groups. Statistical analyses revealed no significant differences between the two groups regarding these characteristics (**Table 1**).

**Table 1:** General demographic information.

Project	BD (n=52)	HC (n=40)	*P*
Age	19.2 (17-22)	20 (17-22)	0.262
Gender,n (%)			0.970
Man	21 (40.40%)	16 (40.00%)	
Female	31 (59.60%)	24 (60.00%)	
BMI	23.02 (19.60-26.40)	22.74 (19.55-24.01)	0.237
Education level,n (%)			0.422
Junior high school	10 (25.00%)	17 (32.69%)	
High school and above	30 (75.00%)	35 (67.31%)	
Smoking	4 (10.00%)	9 (17.30%)	0.319

BD, bipolar disorder; HC, healthy controls; BMI, body mass index.

### 3.2 Detection results of lymphocyte and inflammatory factors

#### 3.2.1 Detection results of lymphocyte subpopulations

The results show that the CD4+ T cell count in the depressed BD patients (median: 957.36 vs. 791.84, P = 0.002) and the proportion of regulatory T cells (Treg%) (median: 6.84% vs. 5.43%, P = 0.003) were significantly higher than in the HC group. The CD56+ NK cell count was significantly higher in the HC group than in the depressed BD patients (median: 422.50 vs. 297.34, *P* = 0.019); although the CD8+ T cell count did not reach the significance threshold (*P* = 0.059), the median in the depressed BD patients group (559.78) was higher than in the HC group (429.83), indicating a potential upward trend. There were no statistically significant differences in CD3+ T cell and CD19+ B cell counts between the two groups (see **Table 2**). The increase in CD4+ T cell count and Treg cell percentage may indicate immune activation or inflammatory status in depressed BD patients, while the decrease in CD56+ NK cells in depressed BD patients may be related to impaired immune surveillance function.

**Table 2:** Comparison of lymphocyte subpopulation detection results between BD and HC groups.

Lymphocyte subpopulations	BD (n=52)	HC (n=40)	*P*
CD3+ TCount	1810.40 ± 511.72	1784.53 ± 655.08	0.832
CD4+T Count	957.36 (822.13-1108.60)	791.84 (537.92-972.85)	0.002
CD8+ TCount	559.78 (304.43-784.27)	429.83 (335.30-483.69)	0.059
CD19+B Count	352.69 ± 142.88	329.22 ± 174.64	0.48
CD56+NK Count	297.34 (211.83-458.57)	422.50 (310.99-523.61)	0.019
Treg %	6.84 (5.09-8.83)	5.43 (4.69-6.71)	0.003

BD, bipolar disorder; HC, healthy controls; CD, cluster of differentiation; Treg, regulatory T cells.

#### 3.2.2 Detection results of inflammatory factors

**Table 3** compares the results of inflammatory factor detection between the BD depression patient and the HC. The median IL-6 in the BD group was 43.86, significantly higher than the 35.62 in the HC group (*P* = 0.020). The median interleukin-8 (IL-8) was 210.13, significantly higher than the 167.55 in the HC group (*P* = 0.012). The median tumor necrosis factor-alpha (TNF-α) was significantly higher than that of the HC group (78.82 vs. 69.15, *P* = 0.029). The median monocyte chemoattractant protein-1 (MCP-1) in the depressed BD patients was 659.22, although higher than the 573.90 in the HC (*P* = 0.095), it did not reach the significance threshold. There were no significant differences in CRP, granulocyte colony-stimulating factor (G-CSF), and CXCL2 between the two groups.

**Table 3:** Comparison of inflammatory factor detection results between BD and HC groups.

Inflammatory factor	BD (n=52) (pg/mL)	HC (n=40) (pg/mL)	*P*
CRP	3189.44 (2337.65-4948.47)	2818.05 (1849.81-4357.08)	0.19
IL6	43.86 (25.87-67.98)	35.62 (24.38-41.57)	0.02
IL8	210.13 (159.69-244.93)	167.55 (130.32-225.35)	0.012
G-CSF	103.25 (87.63-142.50)	103.32 (78.04-124.39)	0.609
MCP-1	659.22 (537.33-843.57)	573.90 (493.59-776.67)	0.095
CXCL2	86.05 (77.71-98.98)	78.87 (60.89-103.11)	0.408
TNF-α	78.82 (64.57-94.96)	69.15 (59.42 -80.81)	0.029

BD, bipolar disorder; HC, healthy controls; CRP, C-reactive protein; IL6, interleukin-6; IL8, interleukin-8; G-CSF, granulocyte colony-stimulating factor; MCP-1, monocyte chemoattractant protein-1; CXCL2, chemokine ligand 2; TNF-α, tumor necrosis factor.

### 3.4 White matter integrity

Compare the FA and MD values of the whole brain white matter (50 ROIs) between depressed BD patients and HC, and analyze the FA and MD values of brain regions with significant differences.

#### 3.4.1 Comparison of brain white matter FA values between depressed BD patients and HC

In the comparison between the depressed BD patients and the HC group, a total of 9 brain regions showed significant differences in FA values (see **Table 4**). Among them, the FA value of Posterior limb of internal capsule R in the BD group averaged 0.41 ± 0.12, significantly lower than the 0.48 ± 0.11 in the HC group (P = 0.009). This suggests that the axonal density or myelin integrity in this region may be compromised, which could be related to motor coordination deficits. The FA values of thalamic-related fiber bundles in BD patients (including Medial lemniscus R and Posterior thalamic radiation R) were significantly lower than those in HC, supporting the critical role of thalamocortical connectivity abnormalities in the pathological mechanisms of depressed BD patients. The median FA value of Sagittal stratum R in the depressed BD patientsgroup was 0.37, significantly lower than the 0.43 in the HC group. The average FA value of Sagittal stratum L in the depressed BD patientsgroup was 0.35 ± 0.12, also significantly lower than the 0.41 ± 0.11 in the HC group. This indicates that the white matter microstructure of the limbic system in depressed BD patients has undergone changes. These changes may affect emotional regulation and cognitive control abilities.

**Table 4:** Brain regions with significant differences in FA values between the BD group and the HC group.

White matter fiber bundle	Fiber bundle coordinates (X,Y,Z)	BD (FA值)	HC (FA值)	*P*
Middle cerebellar peduncle	(69,83,36)	0.28 (0.24-0.35)	0.35 (0.27-0.46)	0.019
Medial lemniscus R	(84,89,40)	0.27 ± 0.13	0.34 ± 0.14	0.017
Cerebral peduncle L	(104,107,60)	0.29 (0.23-0.41)	0.40 (0.26-0.50)	0.032
Posterior limb of internal capsule R	(66,108,85)	0.41 ± 0.12	0.48 ± 0.11	0.009
Retrolenticular part of internal capsule R	(61, 101,78)	0.42 (0.32-0.52)	0.52 (0.42-0.55)	0.013
Posterior thalamic radiation R	(57, 64,73)	0.36 (0.28-0.52)	0.47 (0.37-0.53)	0.046
Sagittal stratum R	(48,97,60)	0.37 (0.25-0.47)	0.43 (0.39-0.48)	0.041
Sagittal stratum L	(131, 97, 60)	0.35 ± 0.12	0.41 ± 0.11	0.038
Cingulum (cingulate gyrus) L	(97, 110, 108)	0.28 ± 0.10	0.32 ± 0.08	0.036

R, right; L, left.

#### 3.4.2 Comparison of brain white matter MD values between depressed BD patients and HC

There are statistically significant differences in the MD values of five brain regions between the BD group and the HC depression (see **Table 5**); In depressed BD patients, the median MD value of the right sagittal stratum was 0.083%, significantly higher than the 0.080% observed in the HC group (P = 0.008), whereas the right fronto-occipital fasciculus showed a median of 0.078% compared to 0.070% in controls (P = 0.026). The median MD value of the left fronto-occipital fasciculus was 0.074%, significantly higher than the 0.072% in the HC group (P = 0.006), and the left uncinate fasciculus showed a median of 0.087% compared to 0.083% in controls (P = 0.044). Additionally, the median MD value of the left superior corona radiata in the depressed BD patients was lower than that in the HC group (P = 0.013). BD depressed patients showed elevated MD values in multiple white matter regions, suggesting damage to the white matter microstructure in this population; whereas the reduced MD values in the left superior corona radiata may indicate a different histopathological process.

**Table 5:** Brain regions with significant differences in MD values between the BD group and the HC group.

White matter fiber bundle	Fiber bundle coordinates (X,Y,Z)	BD (MD值 %)	HC (MD值 %)	*P*
Superior corona radiata L	(116,109,96)	0.073 (0.069-0.088)	0.077 (0.071-0.111)	0.013
Sagittal stratum R	(48,97,60)	0.083 (0.079-0.092)	0.080 (0.077-0.084)	0.008
Superior fronto-occipital fasciculus R	(69, 121, 93)	0.078 (0.069-0,107)	0.070 (0.068-0.083)	0.026
Superior fronto-occipital fasciculus L	(110, 121, 93)	0.074 (0071-0.117)	0.072 (0.068-0.076)	0.006
Uncinate fasciculus L	(123, 125, 58)	0.087 (0.080-0.096)	0.083 (0.078-0.089)	0.044

BD, bipolar disorder; HC, healthy controls; MD, mean diffusion rate; R, right; L, left.

### 3.5 Cognitive function test

The statistical analysis results show that there are significant differences between the depressed BD patients and the HC group in Go/No-Go ACC_diff, ODD-RT_diff, SSRT in the Stop-Signal task, and Stroop ES_RT (P = 0.027, P = 0.04, P = 0.018, P = 0.048). The results of the Go/No-Go task indicate that the inhibitory control ability of depressed BD patients is lower than that of the HC; the results of the More-odd shifting task suggest that the depressed BD patients has deficiencies in flexibility of rule switching and efficiency in handling working memory load. In the Stop-Signal task, the reaction time for signal stop in the depressed BD patients (294.79 ± 35.54) ms is significantly longer than that of the HC group (272.26 ± 53.60) ms, indicating a decline in the BD group’s ability to inhibit initiated actions. In the Stroop task, the median Stroop reaction time effect for BD patients (148.77) is significantly higher than 98.90 (P = 0.048), reflecting deficiencies in conflict monitoring ability among depressed BD patients. However, there are no statistically significant differences in other cognitive function indicators (see **Table 6**).

**Table 6:** Comparison of cognitive function test results between BD group and HC group.

Project	BD (n=52)	HC (n=40)	*P*
Go/No-Go_ACC_diff	0.067 (-0.255-0.190)	0.15 (-0.0722-0.305)	0.027
N-back task ACC	47.83 (25.47-68.43)	49.43 (26.32-72.92)	0.972
N-back task_RT	534.32 ± 16.32	527.38 ± 14.87	0.755
ODD-ACC -Diff	-0.0328 (-0.1132- 0.0204)	-0.0328 (-0.1093- -0.003)	0.622
ODD-RT_Diff	115.17 ± 67.15	85.05 ± 70.82	0.04
Stop-Signal task-SSRT	294.79 ± 35.54	272.26 ± 53.60	0.018
Stroop_ES_ACC	0.02 (0-0.04)	0.02 (0-0.06)	0.484
Stroop_ES_RT	148.77 (59.42-245.12)	98.90 (42.89-179.30)	0.048
SWM_mean_ACC	4.50 (4.50-5.50)	4.50 (3.75-5.50)	0.365

BD, bipolar disorder; HC, healthy controls; Go/No-Go_ACC_Diff, go/no-go accuracy difference; N-back task ACC, N-back task accuracy; N-back task RT, N-back_task reaction time; ODD-ACC -Diff, more-odd shifting accuracy difference; ODD-RT_Diff, more-odd shifting reaction time difference; Stop-Signal task-SSRT, stop-signal task- stop-signal reaction time; Stroop_ES_ACC, stroop effect size accuracy; Stroop_ES_RT, stroop effect size reaction time; SWM_mean_ACC, spatial working memory mean accuracy.

### 3.6 Correlation analysis

#### 3.6.1 Correlation analysis between immune inflammatory markers and FA values in different brain regions

**Figure 1:**
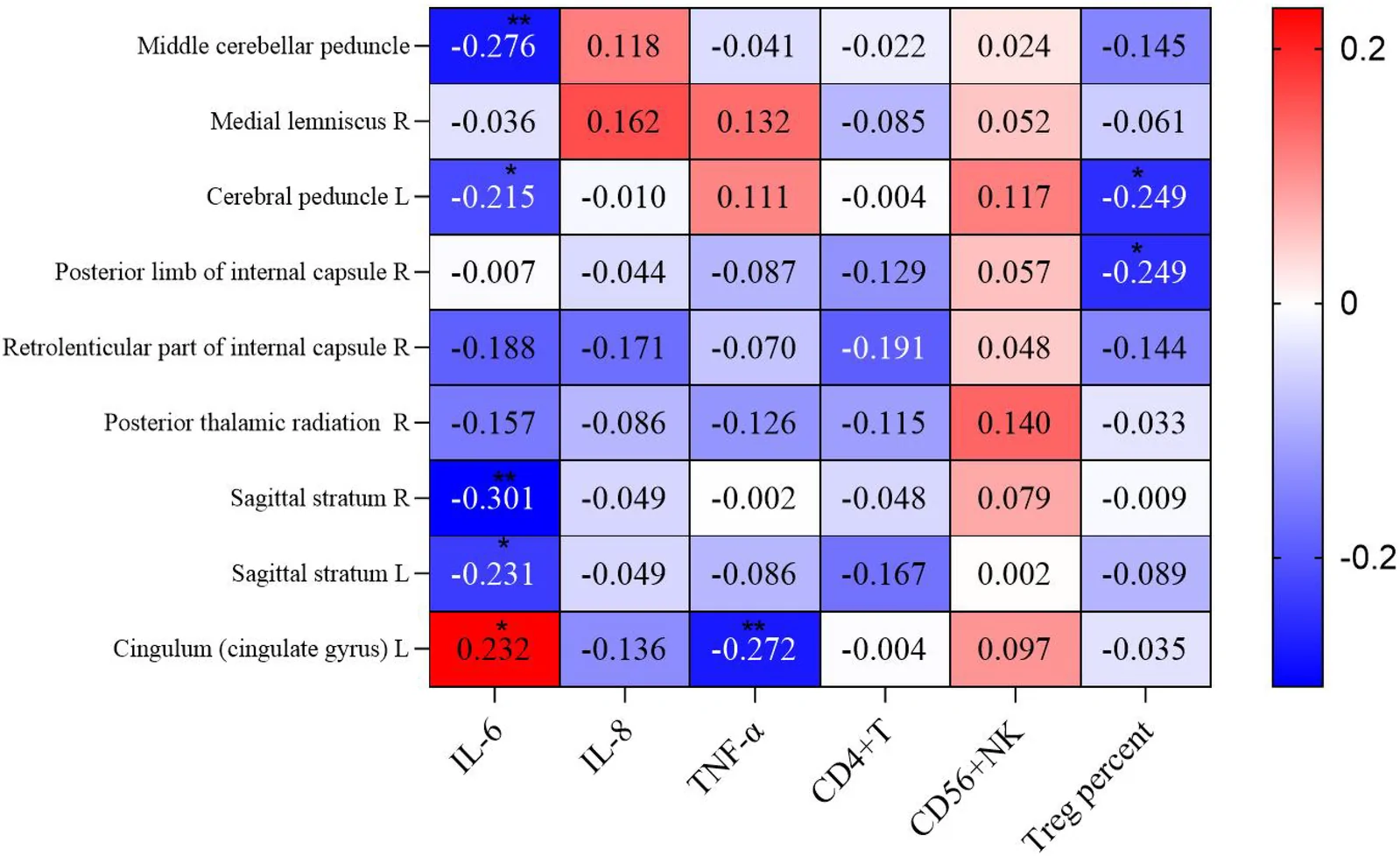
Correlation analysis between inflammatory factors, lymphocyte subpopulations, and FA values in different brain regions(the redder the color, the more representative of both the stronger the positive correlation, the more bluer the color, the more representative of both the stronger the negative correlation, * indicated p < 0.05; ** indicated p < 0.01).

**Figure 1** illustrates that IL-6 exhibited weak to negative correlations with FA values in multiple brain regions, including the middle cerebellar peduncle, left cerebral peduncle, right sagittal stratum, and left sagittal stratum (r ranging from -0.301 to -0.215, p<0.05). Additionally, IL-6 exhibited a weak correlation with FA values in the left cingulate gyrus (r = 0.232, p = 0.027). TNF-α showed a weak negative correlation solely with the left cingulate gyrus (r = -0.272, p = 0.009). The percentage of Treg cells showed identical weak negative correlations with both the left cerebral peduncle and the right posterior limb of the internal capsule (r = -0.249, p = 0.018 for both). IL-6, TNF-α, and the percentage of Treg cells demonstrated statistically significant correlations with FA values in multiple brain regions (p<0.05). However, all significant r values ranged from -0.301 to 0.232, falling within the weak correlation range. Given the small effect sizes, the clinical or neurobiological significance of these associations may be limited.

#### 3.6.2 Correlation analysis between immune inflammatory markers and MD values in different brain regions

**Figure 2:**
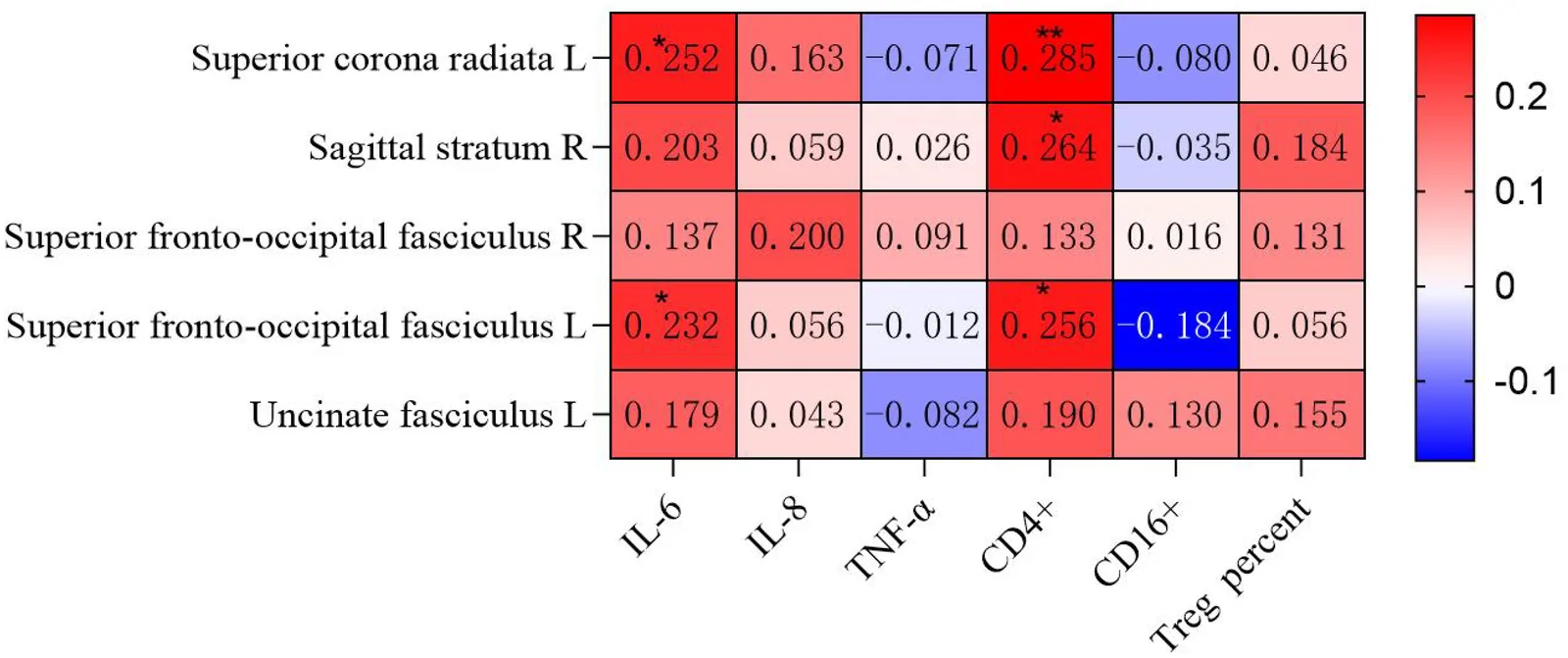
Correlation analysis of inflammatory factors and lymphocytes with the MD values of different brain regions(the redder the color, the more representative of both the stronger the positive correlation, the more bluer the color, the more representative of both the stronger the negative correlation, * indicated p < 0.05; ** indicated p < 0.01).

**Figure 2** shows the correlation between IL-6, IL-8, TNF-α, CD4+ T cell count, CD56+ NK cell count, and the percentage of Treg cells with the MD values of different brain regions. The results indicate IL-6 showed a weak correlation with the left superior corona radiata (r=0.252, P = 0.015) and the left superior fronto-occipital fasciculus (r=0.232, P = 0.026). CD4^+^ T cell count showed a weak correlation with MD values in multiple brain regions, including the left superior corona radiata (r=0.285, P = 0.006) and the right sagittal layer (r=0.264, P = 0.011). The absolute r values of all significant correlations ranged from 0.232 to 0.285, indicating weak correlations. Although the effect size is limited, directionally, higher levels of inflammatory factors (IL-6, CD4^+^ T cells) tend to be associated with increased MD values, which may suggest white matter microstructural damage or edema, these results are only considered exploratory findings.

#### 3.6.3 Analysis of the correlation between immune inflammation and cognitive function

**Figure 3:**
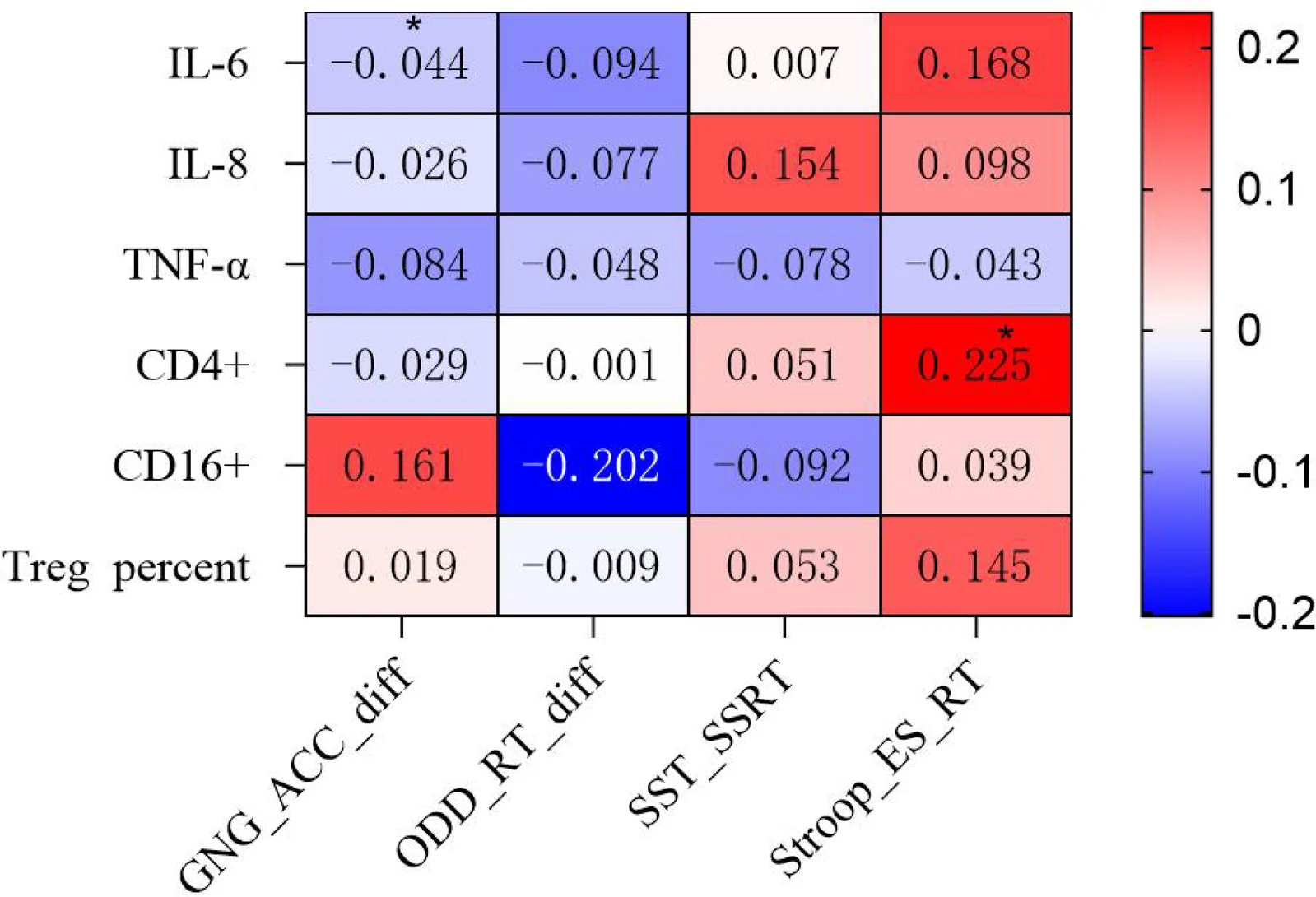
Correlation Analysis between Immune Inflammation and Cognitive Function (the redder the color, the more representative of both the stronger the positive correlation, the more bluer the color, the more representative of both the stronger the negative correlation, * indicated p < 0.05).

**Figure 3** shows the correlation between IL-6, IL-8, TNF-α, CD4+ T cell count, CD56+ NK cell count, Treg cell percentage, and cognitive function. IL-6 was negatively correlated with the difference in inhibitory control ability (GNG_ACC_diff) (r=-0.044, P = 0.027), while CD4+ T cell count was positively correlated with the Stroop conflict effect (Stroop_ES_RT) (r=0.225, P = 0.032), suggesting that individuals with higher CD4+ T cell levels may exhibit a slight increase in the Stroop interference effect. However, given the small effect size, this finding is considered only a preliminary exploratory observation.

#### 3.6.4 Analysis of the correlation between FA values in different brain regions and cognitive function

**Figure 4:**
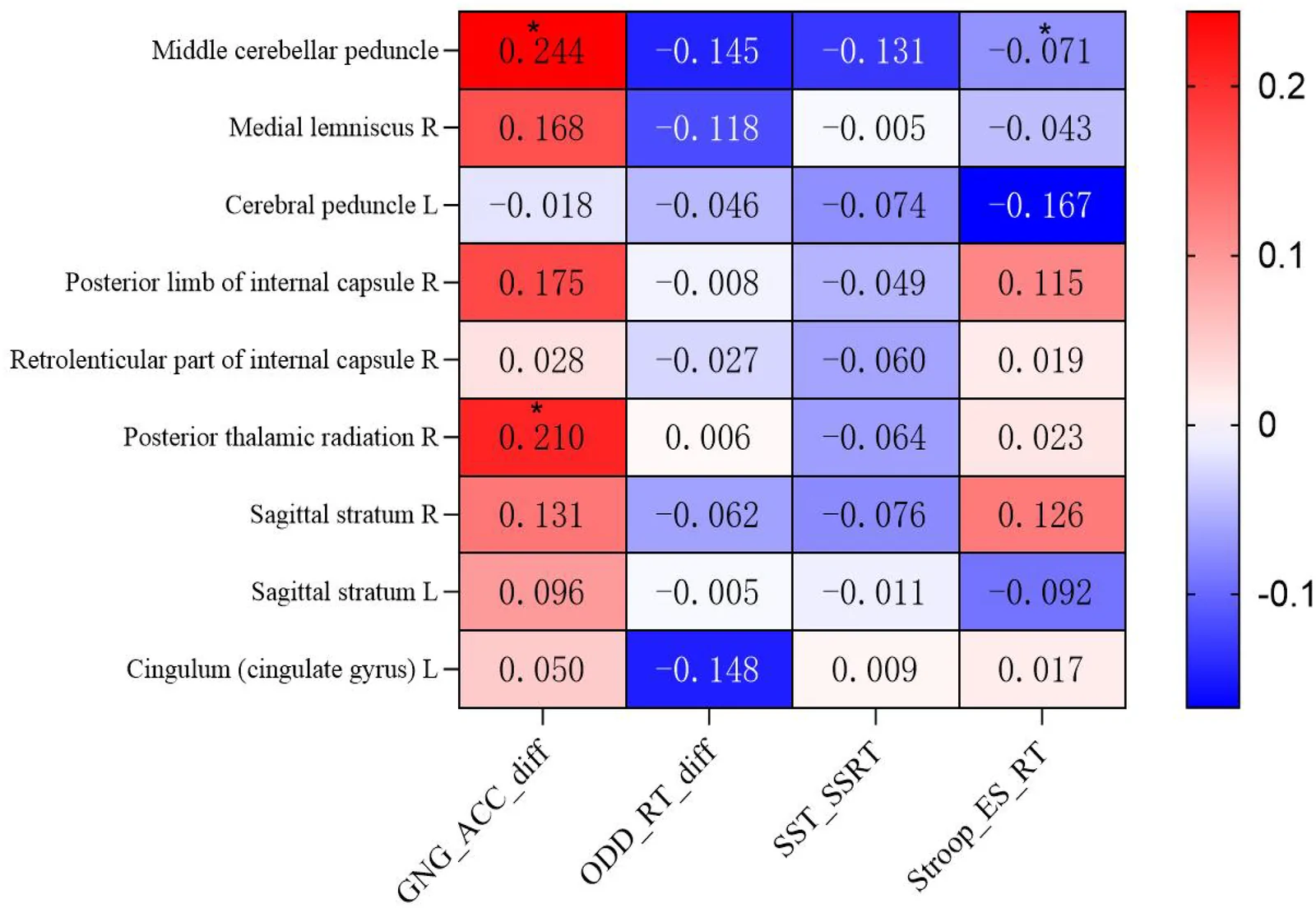
Correlation analysis between FA values of different brain regions and cognitive function the redder (the color, themorerepresentative of both the stronger the positive correlation, the more bluer the color, the more representative of both the stronger the negative correlation, * indicated p < 0.05).

**Figure 4** shows the correlation between FA values in different brain regions and cognitive function. The results showed that the middle cerebellar peduncle and the right posterior thalamic radiation exhibited a weak positive correlation with Go/No-Go_ACC_diff (middle cerebellar peduncle: r=0.244, P = 0.019; right posterior thalamic radiation: r=0.210, P = 0.045); the middle cerebellar peduncle demonstrated a very weak negative correlation with Stroop_ES_RT (r=-0.071, P = 0.043). These results suggest that higher FA values in the middle cerebellar peduncle and the right posterior thalamic radiation may be weakly associated with enhanced inhibitory control, although the observed effect sizes were small.

#### 3.6.5 Analysis of the correlation between MD values in different brain regions and cognitive function

**Figure 5:**
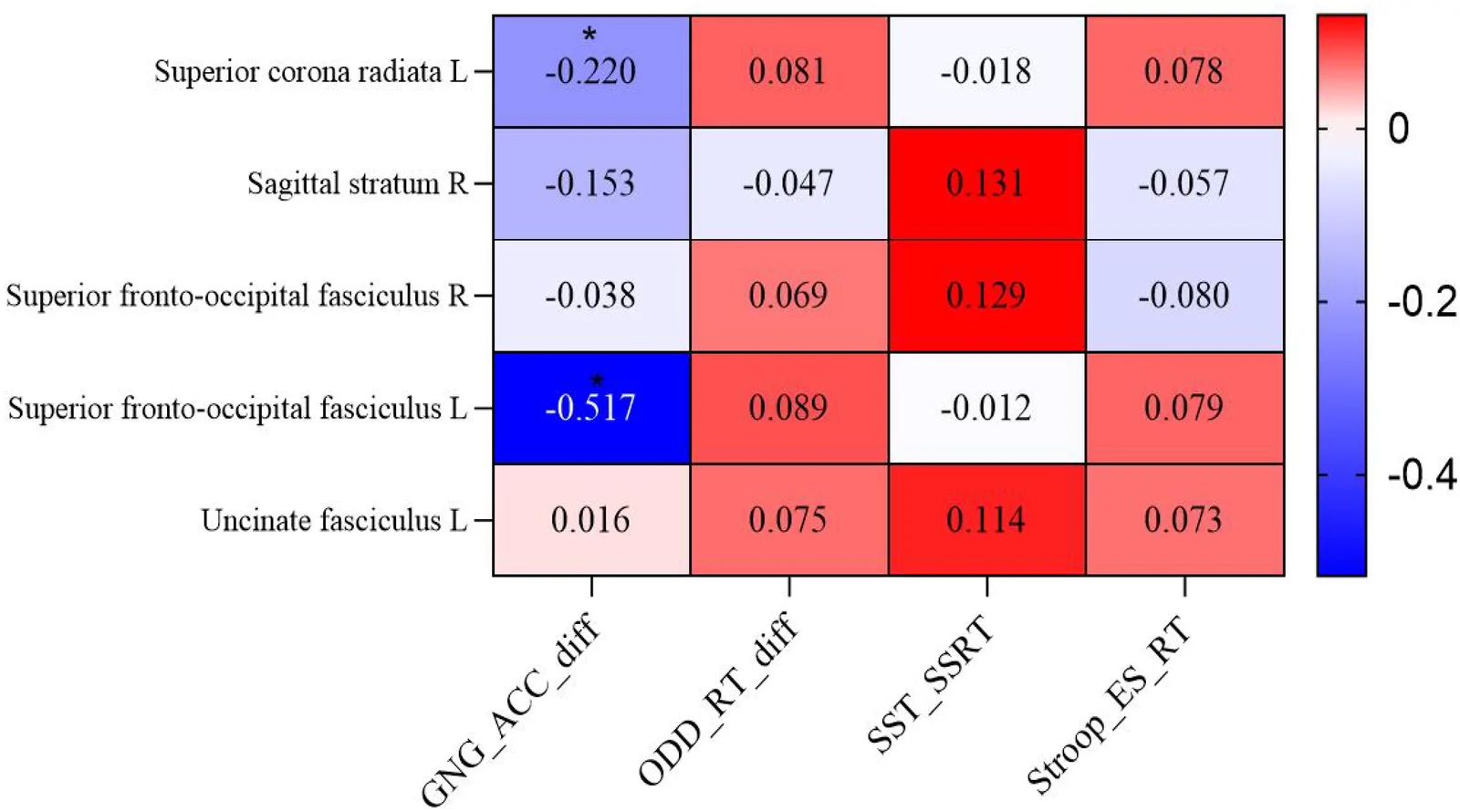
Correlation analysis between MD values of different brain regions and cognitive function (the redder the color, themorerepresentative of both the stronger the positive correlation, the more bluer the color, the more representative of both the stronger the negative correlation, * indicated p < 0.05).

**Figure 5** shows the correlation between the MD values of different brain regions and four cognitive functions. The results indicate that the MD in the left superior corona radiata showed a weak negative correlation with Go/No-Go_ACC_diff (r = -0.220); MD in the left superior fronto-occipital fasciculus was negatively correlated with Go/No-Go_ACC_diff (r = -0.517), indicating that elevated MD in this region (possibly reflecting white matter microstructural damage) is significantly associated with a decline in inhibitory control.

### 3.7 Machine learning model analysis

**Figure 6:**
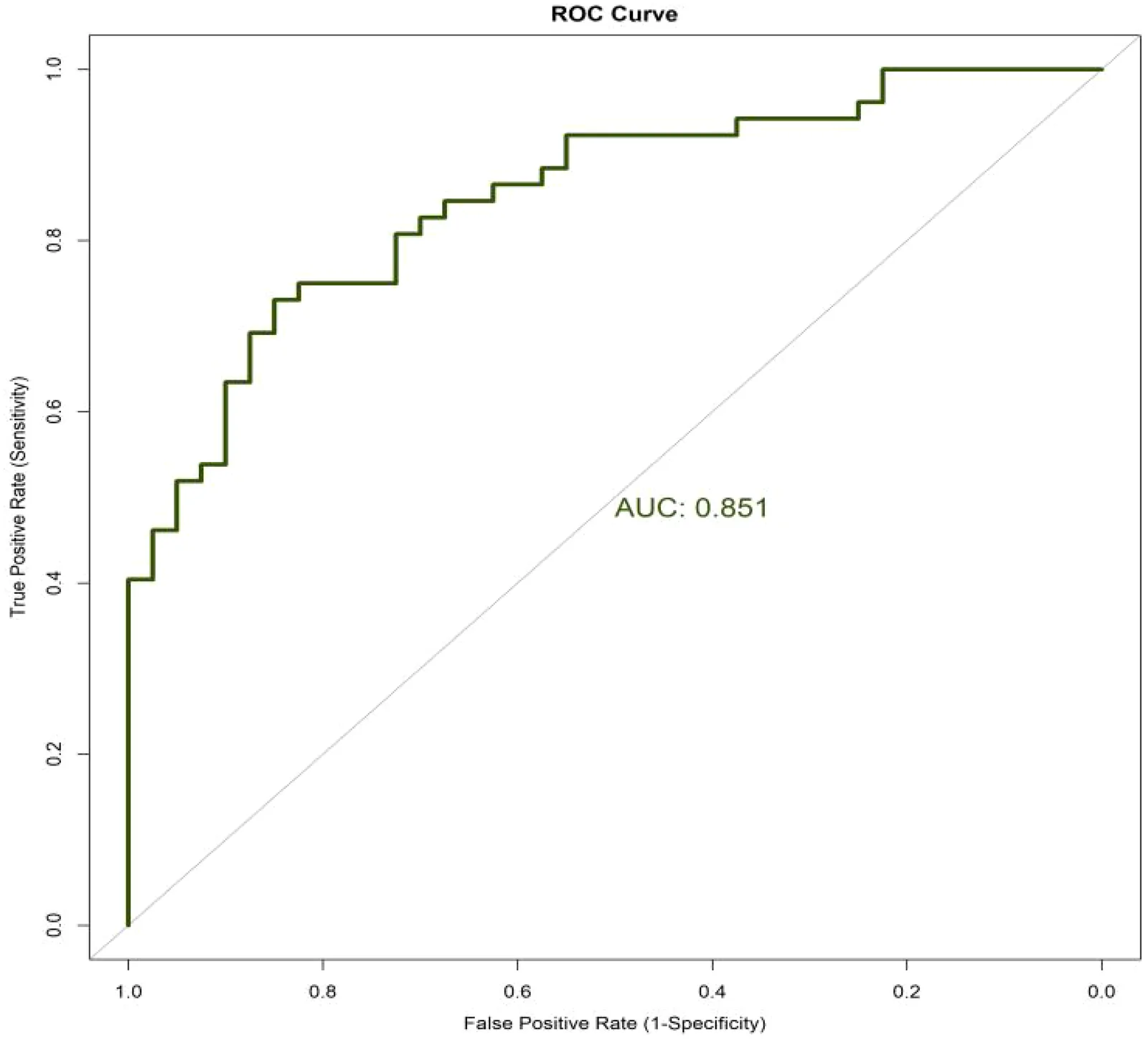
ROC curve analysis of the Support Vector Machine (SVM) model.

The Support Vector Machine (SVM) model demonstrated robust classification performance in distinguishing depressed BD patients from HC, with an overall accuracy of 76.09%, sensitivity of 82.69%, and specificity of 67.50% (**Figure 6**). The area under the curve (AUC) obtained through Receiver Operating Characteristic (ROC) analysis was 0.851, indicating good discriminative ability of the model. By analyzing feature weights, we identified the biomarkers that contributed most to the classification. Specifically, performance on the Stroop task (Stroop_ES_RT) (β=1.155 ± 0.056), levels of the inflammatory factor IL-6 (β=1.073 ± 0.069), and concentrations of TNF-α (β=1.071 ± 0.062) emerged as the most discriminative features. Regarding white matter microstructure, fractional anisotropy (FA) values in the right medial thalamic tract (β=0.758 ± 0.047) and the right posterior thalamic radiation (β=0.756 ± 0.068), alongside the immune cell subgroup CD56+NK (β=0.744 ± 0.052), exhibited significant predictive value. These results suggest that changes in executive function, neuroinflammation, and white matter microstructure may collectively contribute to the pathological process of depressed BD patients. Moreover, this panel of biomarkers offers a promising foundation for the clinical diagnosis of depressed BD patients.

BD.

## Discussion

Cognitive dysfunction in patients with BD exerts a profound impact, not only impairing daily psychosocial functioning but also affecting core disease outcomes (5). Even during euthymic periods, adolescents with BD exhibit significant cognitive dysfunction, characterized by impairments in verbal learning, memory, working memory, and visual learning (30) Further investigation into cognitive impairment in patients with BD holds the potential to improve their quality of life and disease outcomes. This study aims to identify biomarkers of cognitive function in BD by integrating immune-inflammatory indicators with magnetic resonance imaging (MRI).

This study conducted a multidimensional analysis of 52 depressed BD patients and 40 HC, examining differences in assessments of lymphocyte subsets and inflammatory cytokines, DTI, and standardized cognitive function tests. The results showed that immune cell counts and levels of inflammatory cytokines in depressed BD patients were significantly higher than those in HC, suggesting a pronounced systemic inflammatory response. Furthermore, significant differences in FA and MD values across multiple brain regions were observed between depressed BD patients and HC, with depressed BD patients performing significantly worse than HC on standardized cognitive function tests. The aforementioned immune-inflammatory indicators, as well as FA and MD values, may serve as potential biomarkers for BD, providing new insights into its pathological mechanisms. These findings not only provide a scientific basis for personalized treatment strategies but also deepen the understanding of the immune-inflammatory mechanisms of BD and the accompanying pathological processes of brain structural abnormalities. Correlation analysis revealed potential links between immune-inflammatory responses, white matter microstructural changes, and cognitive impairment.

This study found significant differences in immune cell subsets between depressed BD patients and HC. Specifically, the bipolar depression group exhibited significantly elevated CD4+T cell counts and regulatory T cell percentages, alongside decreased CD56+NK cell counts, indicating alterations in circulating immune cell distribution. Previous studies have demonstrated significant differences in T lymphocyte subpopulations among patients with BD, suggesting that immune cells play a role in the development and progression of mood disorders (31–33). The compensatory immune regulatory system is implicated in the pathophysiology of mood disorders by modulating primary immuno-inflammatory responses, potentially promoting spontaneous recovery and enhancing antidepressant efficacy (34), While previous research indicates that the number of HLA-DR-expressing NK cells is increased yet functionally impaired in BD patients (35), the current study observed a reduction in CD56+ NK cell counts, suggesting a distinct immunopathological mechanism; changes in lymphocyte subsets support the chronic inflammatory characteristics of BD (33), elucidating the role of immuno-inflammation in the pathophysiology of bipolar depression and suggesting the potential application of novel anti-inflammatory therapies.

White matter integrity analysis revealed that depressed BD patients exhibited significantly lower FA values in multiple brain regions compared to HC, particularly in key areas such as the right posterior limb of the internal capsule, thalamus-related fiber tracts, and the left cingulate gyrus. Additionally, the BD group demonstrated significantly elevated MD values in multiple brain regions relative to the control group. Studies indicate that reduced FA values in the corpus callosum may impair motor coordination and emotion regulation (18, 36). The elevated MD values support the hypothesis of chronic white matter degenerative changes and provide important evidence for elucidating the pathological mechanism underlying mood fluctuations in BD (36). White matter microstructural damage may impair emotion regulation, particularly in limbic system regions such as the cingulate gyrus and prefrontal cortex (37). The observed compromised white matter integrity in depressed BD patients, although these differences are statistically significant, the absolute changes are minimal, yet these subtle microstructural alterations may reflect early or subthreshold pathological processes, rather than act as reliable diagnostic markers.

In cognitive function tests, we found that patients with depressed BD patients exhibited significant executive function deficits, particularly in response inhibition and task switching, performing worse than HC on the Go/No-Go and Stroop tasks. Cognitive function deficits may be related to impaired emotion regulation and decision-making abilities in BD patients, and such patients show impairments in attention, memory, and executive function during both the acute phase and remission period (38). The decline in response inhibition is a key cognitive impairment in BD, leading to increased impulsivity in BD patients (39). This finding is consistent with the results of this study. Cognitive impairment may reduce or increase the risk of neurodegenerative diseases (40). Improving cognitive impairment is a key goal alongside symptom remission in the treatment of BD.

The correlation analysis of this study revealed that IL-6 exhibited only a weak negative correlation with FA values in brain regions, and TNF-α showed only a weak negative correlation with FA values in the left cingulate gyrus, suggesting a limited association between inflammatory factors and white matter microstructure. Previous studies have reported an inverse correlation between tumor necrosis factor-α (TNF-α) levels and white matter integrity in patients with BD (12), research has shown that abnormalities in white matter fibers in BD patients are highly correlated with a reduction in circulating T-cell subsets (41). At the same time, TBSS studies show that the microstructure of white matter in BD patients is widely altered, with FA negatively correlated and radial dispersion degree (RD) and CD4+ T cells positively correlated (42). The weak correlations in this study may be attributable to sample size or confounding variables (such as age, disease duration, and prior medication use). Therefore, future studies with larger samples, including animal experiments, are needed to further verify whether immune-inflammatory changes cause white matter abnormalities in patients with BD.

Further analysis revealed that elevated IL-6 levels showed a weak positive correlation with reduced inhibitory control ability, and CD4+ T cell count also exhibited a weak positive correlation with the Stroop conflict effect. Both findings indicate that inflammation and immune activation are associated with poorer cognitive performance, albeit with modest effect sizes. Various immunologic factors consistently demonstrated associations in the same direction, suggesting the broad nature of immune-cognitive interactions in BD. Although the correlations are weak, the results are consistent with previous studies, supporting the regulatory role of immune-inflammatory responses on executive function through their effects on neuroplasticity and white matter integrity (43).

Previous studies suggest that the mechanisms of cognitive impairment are potentially associated with various factors such as genetic polymorphism, brain structural and functional variables, inflammation, and metabolic factors (38). Changes in white matter microstructure may serve as the biological basis for cognitive dysfunction in patients with BD (44). This study demonstrates that lower FA values in certain brain regions exhibit a weak correlation with decreased inhibitory control and prolonged reaction time in the Stroop task, supporting the established link between white matter integrity and cognitive function. Mechanistically, the study suggests a cascade of damage along the “immune-white matter-cognitive function axis” during the depressive phase of BD: chronic inflammation precipitates disruptions in white matter microstructure, which in turn impairs executive functions (such as inhibitory control and conflict processing). However, since the association between white matter and cognition remains modest, this pathway warrants further validation with larger samples and longitudinal studies. Future intervention strategies may need to integrate anti-inflammatory therapy and cognitive rehabilitation to achieve multi-target modulation, but the limitations of the current association strength should be fully considered in clinical translation.

Finally, the support vector machine (SVM) model we constructed demonstrated robust classification performance based on these immune inflammation markers, abnormal neuroimaging features, and cognitive functions in distinguishing BD patients from HC, achieving an accuracy of 76.09%. Feature analysis showed that changes in executive function, inflammatory factors, and white matter microstructure may jointly contribute to the pathological process of bipolar depression, providing a potential combination of biomarkers for the diagnosis of the disorder. In addition, the biomarkers identified through feature weight analysis, such as IL-6 and TNF-α, further support the importance of immune inflammation in the pathological mechanisms of bipolar depression. These results offer novel insights for future personalized treatment, emphasizing that targeting the regulation of the immune system in clinical interventions may help improve cognitive function and overall quality of life in depressed BD patients.

This study further suggests a weak correlation between neuroinflammation, white matter abnormalities, and cognitive function. Consequently, these findings tentatively suggest a potential “immune-white matter-cognition function axis” cascade damage mechanism during the depressive phase of bipolar depression: chronic inflammatory states may lead to white matter microstructural degeneration, thereby contributing to mild impairment in executive functions such as inhibitory control and conflict processing. Given the modest magnitude of these associations, this mechanistic hypothesis requires validation through larger samples, longitudinal designs, or mediation analyses. Future intervention strategies for depressed BD patients could potentially explore combining anti-inflammatory therapy with cognitive rehabilitation, though clinical translation should be approached with caution.

This study offers insights into the changes in white matter microstructure, cognitive function, and their relationship with the immuno-inflammatory response in patients experiencing depressive episodes of bipolar disorder (BD); however, several limitations remain: (1) The limited sample size and heterogeneity within the enrolled population may compromise the generalizability and statistical power of the results. (2) The absence of longitudinal data constitutes a limitation; as this study employs a cross-sectional design, it cannot capture the dynamic trajectories of the response indicators. (3) Potential confounding factors remain, as unmeasured variables may have influenced the results. (4) Fourth, the mechanistic exploration in this study lacks sufficient depth. Future research should combine molecular biology and neuroimaging techniques to explore the biological mechanisms behind these changes in depth. (5) The findings of this study are restricted to untreated patients with BD depression or those in the relatively acute phase; therefore, they cannot be directly extrapolated to patients receiving maintenance treatment. (6) Future studies should incorporate patient subgroups stratified by treatment status to comprehensively evaluate the neuroimaging features of BD depression. (7) The SVM model is based on a small sample of 92 patients and incorporates 19 predictive features. The ratio of features to sample size (approximately 1:4.8) falls within a critical range, posing a risk of model overfitting. In the future, a larger sample is needed to further support the reliability of the model.

## Conclusion

Inflammation and impaired WM integrity may offer a scientific basis for understanding the potential mechanisms of depressed BD patients Based on the observed weak correlations, this study can only preliminarily speculate that chronic inflammation and white matter microstructural abnormalities may have an impact on cognitive function.

## Data Availability

The original contributions presented in the study are included in the article/supplementary material. Further inquiries can be directed to the corresponding authors.
