# Correction: Impact of immune function and white matter integrity on cognitive function in patients with bipolar disorder

**DOI:** 10.3389/fimmu.2026.1959378

**Published:** 2026-08-20

**Authors:** Zhipeng Wang, Tingting Xie, Yumei Wang, Xihua Jia, Xiaobo Wang

**Affiliations:** 1 Department of Ultrasound, the Third Medical Center of Chinese PLA General Hospital, Beijing, China; 2 Institute of Brain Science and Brain-Inspired Research, Shandong First Medical University &Shandong Academy of Medical Sciences, Jinan, Shandong, China; 3 Departmentof Psychology, Shandong Provincial Hospital Affiliated to Shandong First Medical University, Jinan, Shandong, China; 4 Department of Oncology, The First Central Hospital of Baoding, Baoding, Hebei, China; 5 Department of Clinical Psychology, The First Central Hospital of Baoding, Baoding, Hebei, China

**Keywords:** bipolar disorder, cognitive function, inflammatory factors, lymphocyte subpopulations, white matter structure

Author “Yumei Wang” was erroneously included as corresponding author. The correct corresponding author(s) is solely “Xiabo Wang”.

The author list has been incorrectly published as “Zhipeng Wang†, Xiaobo Wang*, Tingting Xie†, Yumei Wang* and Xihua Jia”, the correct order should have been the following:

“Zhipeng Wang†, Tingting Xie†, Yumei Wang, Xihua Jia and Xiaobo Wang*”

The original version of this article has been updated.

